# Draft Genome Sequence of *Mycobacterium wolinskyi*, a Rapid-Growing Species of Nontuberculous Mycobacteria

**DOI:** 10.1128/genomeA.00138-16

**Published:** 2016-03-17

**Authors:** Tom J. B. de Man, K. Allison Perry, Adrian Lawsin, Angela D. Coulliette, Bette Jensen, Nadege C. Toney, Brandi M. Limbago, Judith Noble-Wang

**Affiliations:** a Division of Healthcare Quality Promotion, Centers for Disease Control and Prevention, Atlanta, Georgia, USA; b Division of Viral Diseases, Centers for Disease Control and Prevention, Atlanta, Georgia, USA

## Abstract

*Mycobacterium wolinskyi* is a nonpigmented, rapidly growing nontuberculous mycobacterium species that is associated with bacteremia, peritonitis, infections associated with implants/prostheses, and skin and soft tissue infections often following surgical procedures in humans. Here, we report the first functionally annotated draft genome sequence of *M. wolinskyi* CDC_01.

## GENOME ANNOUNCEMENT

Nontuberculous mycobacteria (NTM) are environmentally ubiquitous and can be a source of both colonization and infection in humans (1). *Mycobacterium wolinskyi* is an uncommon, rapid growing NTM previously belonging to the *Mycobacterium smegmatis* group (2). Although fewer than 30 clinical cases have been described worldwide, *M. wolinskyi* is associated with bacteremia (3, 4), peritonitis (5), infections associated with implants/prostheses (6–8), and postsurgical skin and soft tissue infections (9–11).

During the course of an outbreak investigation of surgical site infections, *M. wolinskyi* was cultured from an environmental water sample and compared to epidemiologically linked patient isolates. All isolates were closely related (91% similar) by pulsed-field gel electrophoresis (PFGE), and differed by 13 to 35 single nucleotide polymorphisms in the core genome. One isolate, *M. wolinskyi* CDC_01, obtained from a surgical site hip wound was selected for functional annotation described here.

DNA was extracted using the Maxwell 16 (Promega, Madison, WI) instrument and the Maxwell 16 cell LEV DNA purification kit from a 72 h culture grown on Middlebrook 7H10. Whole-genome sequencing (WGS) was performed using a MiSeq (Illumina, San Diego, CA) and generated paired-end reads of 251 bp with 47-fold coverage on average. Prior to our analysis we screened out viral contaminants from our raw sequencing reads using Bowtie2 (12) and the RefSeq viral database (release 72).

Subsequently, k-mers (*k* = 31) were counted from the virus free sequencing reads with Jellyfish (13) and the observed frequency and volume were used as an assembly-independent method to estimate the true genome size (14). Using this method, we determined the genome size as being close to 7.23 Mbp.

Sequencing reads were then trimmed and adapters removed by means of fastq-mcf (http://code.google.com/p/ea-utils) and subsequent *de novo* assembly was performed using spades 3.1 (15). Contigs smaller than 500 bp were discarded using a custom Perl script. The final filtered assembly consists of 76 contigs with a total length of 7,449,739 bp and an *N*_50_ size of 221,566 bp. The G+C content is 66.52%, within the expected range for *Mycobacterium* species (16–18). In order to determine the purity of our genome assembly, contigs were screened against the RefSeq bacterial genome database (release 72) by means of KRAKEN (19). All sequences mapped to species belonging to the order of *Actinomycetales*, which includes *M. wolinskyi*. Since this is the first sequenced genome of *M. wolinskyi*, no contigs mapped specifically to this species. The largest proportion of contigs (*n* = 40; 53%) mapped to *M. smegmatis*, indicating it is closely related to *M. wolinskyi* (2).

Contig annotation yielded 6,901 protein-coding genes, 5 rRNA genes (5S, 16S, and 23S), and 71 tRNAs representing all 20 amino acids according to the NCBI Prokaryotic Genome Annotation Pipeline (PGAP). Additionally, the genome assembly was searched using SSTAR v1.0 (20) and the ARG-ANNOT database (21); however, no acquired or intrinsic genes encoding antibiotic resistance were detected.

This *M. wolinskyi* draft genome assembly will aid in future clinical outbreaks as a reference and will help elucidate the genomic features of a human pathogen.

### Nucleotide sequence accession numbers.

This whole-genome shotgun project has been deposited at DDBJ/EMBL/GenBank under the accession no. LGTW00000000. The version described in this paper is version LGTW01000000.
